# PET Imaging for Initial Staging and Therapy Assessment in Multiple Myeloma Patients

**DOI:** 10.3390/ijms18020445

**Published:** 2017-02-18

**Authors:** Clément Bailly, Rodolphe Leforestier, Bastien Jamet, Thomas Carlier, Mickael Bourgeois, François Guérard, Cyrille Touzeau, Philippe Moreau, Michel Chérel, Françoise Kraeber-Bodéré, Caroline Bodet-Milin

**Affiliations:** 1 Nantes-Angers Cancer Research Center (CRCNA), University of Nantes, Inserm UMR 1232, 8 quai Moncousu, 44007 Nantes, France; clement.bailly@chu-nantes.fr (C.B.); thomas.carlier@chu-nantes.fr (T.C.); mickael.bourgeois@nantes.inserm.fr (M.B.); francois.guerard@univ-nantes.fr (F.G.); michel.cherel@univ-nantes.fr (M.C.); francoise.bodere@chu-nantes.fr (F.K.-B.); 2 Department of Nuclear Medicine, CHU de Nantes, 1 place Alexis Ricordeau, 44093 Nantes, France; rodolpheleforestier@hotmail.fr (R.L.); bastien.jamet@chu-nantes.fr (B.J.); cyrille.touzeau@chu-nantes.fr (C.T.); philippe.moreau@chu-nantes.fr (P.M.); 3 Department of Nuclear Medicine, ICO-René Gauducheau, Boulevard Jacques Monod, 44805 Saint-Herblain, France

**Keywords:** multiple myeloma, solitary plasmacytoma, PET/CT, therapeutic evaluation

## Abstract

Multiple myeloma (MM) is a hematological neoplasm characterized by the clonal proliferation of malignant plasma cells in the bone marrow. MM results in diffuse or focal bone infiltration and extramedullary lesions. Over the past two decades, advances have been made with regard to the diagnosis, staging, treatment, and imaging of MM. Computed tomography (CT) and magnetic resonance imaging (MRI) are currently recommended as the most effective imaging modalities at diagnostic. Yet, recent data from the literature suggest that positron emission tomography combined with computed tomography (PET/CT) using ^18^F-deoxyglucose (FDG) is a promising technique for initial staging and therapeutic monitoring in this pathology. This paper reviews the recent advances as well as the potential place of a more specific radiopharmaceutical in MM.

## 1. Introduction

Multiple myeloma (MM) is a malignancy characterized by the clonal proliferation of plasma cells. It is marked by heterogeneous phenotypic, genetic and clinical presentation and it is almost always preceded by monoclonal gammopathy of undetermined significance (MGUS) [1]. Smoldering multiple myeloma (SMM) represents a mid-clinical stage between MGUS and MM. This latter constitutes a heterogeneous entity including patients displaying a very slow progression towards an identified MM within several years and patients progressing rapidly towards symptomatic myeloma (high-risk SMM).

The definition of symptomatic MM, a clinical staging requiring treatment, was traditionally based on the presence of organ damage related to plasma cell growth as defined by CRAB criteria (hypercalcemia, renal insufficiency, anemia and the presence of bone lesions). This definition was revised in 2014 by the International Myeloma Working Group (IMWG), integrating new prognostic biomarkers, with the aim of not delaying the initiation of treatment of high-risk SMM-classified patients and to avoid the establishment of harmful bone lesions or renal impairment [2]. New biomarkers have therefore been defined as associated with an 80% probability of progression towards positive MM CRAB criteria within two years, making it possible to identify patients requiring therapy: clonal bone marrow plasma cell percentage ≥60%, involved/uninvolved serum free light chain ratio ≥100, and more than 1 focal bone lesion (FL) (≥5 mm in size) on magnetic resonance imaging (MRI) studies.

Given that the presence of even an asymptomatic bone disease must be considered as a treatment criterion, imaging plays a significant role in the management of MM [2]. Although a standard skeletal survey was traditionally considered as the reference technique, studies carried out over the last 10 years have established the superior performances of low-dose whole-body computed tomography (CT-WB) and MRI. Positron emission tomography (PET) using ^18^F-deoxyglucose (FDG-PET), a marker of glucose metabolism, produces performances similar to that of morphological imaging techniques in bone lesions’ detection. MRI detects bone abnormalities in more than 90% of patients presenting with symptomatic MM and appears as the best procedure for evaluating painful lesions and detecting medullary compression [3]. On the other hand, in the therapeutic follow-up, the MRI performances are less satisfactory due to a high frequency of false-positive images, while FDG-PET appears to be more effective [2,3]. In addition, MRI is recommended in SMM patients: patients presenting more than 1 non-equivocal FL (diameter >5 mm) must be considered as suffering from symptomatic MM and requiring treatment [2,3]. In patients presenting equivocal anomalies in MRI, staging can be completed by a CT-WB or a FDG-PET to confirm asymptomatic bone impairment [2].

By detecting tumor cells or a tumor environment with high glucose consumption, FDG-PET provides additional information to that provided by MRI or CT [2]. In criteria revised in 2014, the detection of one or more osteolytic lesions in FDG-PET defines MM bone disease recognized by the CRAB criteria. FDG-PET can also be proposed to patients with SMM if MRI is inaccessible or non-conclusive [2]. In addition, FDG-PET allows for the detection of extra-medullary disease (EMD) and provides prognostic information for symptomatic MM at baseline and therapeutic follow-up [4,5,6,7]. FDG-PET is of equal interest for patients with solitary plasmacytoma (SP) to detect EMD [8,9] and has a prognostic value in patients with SMM [10,11].

Other radiopharmaceuticals targeting alternative MM biomarkers have also shown promising results, such as radio-labeled choline, ^68^Ga-Pentixafor targeting C–X–C chemokine receptor type 4 (CXCR4), and immuno-PET using radiolabeled monoclonal antibodies (mAbs) as a companion of antibody-based therapies [12,13,14,15,16].

## 2. FDG-PET for Bone Disease Detection

Despite some variations from one study to another, FDG-PET permits a whole-body investigation with an overall sensitivity of 90% and specificity of 75% for the detection of myeloma lesions [5,6,7]. It has the ability to show diffuse involvement, FLs, or mixed bone diseases with variable glucose uptake, resulting in heterogeneous maximum standardized uptake values (SUVmax). PET-FDG enables the detection of EMD, which occurs in less than 10% of patients at diagnosis. FDG-PET is also useful for evaluating patients with non- or slightly secreting forms of myeloma, which cannot be evaluated by biological methods.

FDG-PET sensitivity is better than a whole-body skeletal survey, showing additional lesions in half of the studied patients yet with reported false negative scans for small size lesions of the skull [17]. The French IMAgerie JEune Myélome (IMAJEM) study has compared FDG-PET and MRI performed at baseline in patients with symptomatic MM, showing comparable results with both techniques, detecting abnormalities in more than 90% of the patients [18]. FDG-PET also allows for the detection of additional medullary lesions or EMD in regions unexplored by MRI. In patients with SP, FDG-PET detects additional lesions with a greater sensitivity and specificity than MRI [8]. In addition, it has been demonstrated that the presence of at least two hypermetabolic lesions predict a rapid progression towards MM [9].

## 3. Prognostic Value of FDG-PET in Baseline Evaluation of Symptomatic MM and SMM

The prognostic value of FDG-PET and MRI were firstly compared in a large prospective series of 239 patients who underwent homogeneous first line treatment in a double autograft program [19]. In multivariate analysis, the only diagnostic imaging modality significantly associated with an unfavorable prognostic value, both for overall survival (OS) and event-free survival (EFS), was FDG-PET when the number of FLs was greater than three at diagnosis. The number of FLs on the baseline MRI (7 and more) affected EFS, but not OS. 

The prognostic value of the number of FLs on FDG-PET at baseline was then confirmed in a large series of 192 patients with MM treated with thalidomide-dexamethasone induction therapy and double autologous stem cell transplantation (ASCT) [20]. In this study, at least 3 FLs (44% of cases), a SUVmax >4.2 (46% of cases) and the presence of EMD (6%) negatively affected four-year progression-free survival (PFS). The SUVmax >4.2 and the presence of EMD were also associated with shorter OS.

The prognostic value of FDG-PET at diagnosis has also been studied in a smaller series of 61 patients including MM (55 patients) or SP (6 patients) [21]. A correlation has been found between the most intense EMD FDG-uptake and both osteo-medullary fixation (*p* = 0.027) and the International Staging System (ISS) score (*p* = 0.048). The bone marrow SUVmax was correlated with the ISS score (*p* = 0.013). The 44 patients with positive FDG-PET had a shorter five-year survival (61%) than the 11 patients with negative FDG-PET patients, all of whom were alive after five years (*p* = 0.01). In multivariate analysis, only the EMD with the highest SUVmax had a prognostic value on OS (*p* = 0.03).

Another study comparing FDG-PET and MRI in a small series of 33 patients with MM at diagnosis concluded that FDG-PET had a prognostic value above MRI [22]. The univariate and multivariate analyses showed that FLs and diffuse bone marrow impairments on FDG-PET affected patients’ PFS (*p* < 0.001), whereas OS was only affected by FLs (*p* = 0.001). The MRI data were not predictive in multivariate analysis. 

Several studies also evaluated the prognostic value of baseline volume-based FDG-PET parameters. These metrics such as metabolic tumor volume (MTV) and total lesion glycolysis (TLG) appear as promising tools by quantifying functional disease burden in MM [23,24]. In a recent study performed with 192 MM patients, baseline TLG higher than 620g and MTV higher than 210 cm^3^ were associated with poor PFS and OS after adjusting for baseline myeloma variables. Combined with the 70-gene expression profiling (GEP70) risk score, TLG higher than 205 g identified a high-risk subgroup, and divided ISS stage II patients into two subgroups with similar outcomes to ISS stage I and ISS stage III. However, because of heterogeneous data, further prospective clinical studies are mandatory to confirm the validity of these results.

In SMM, FDG-PET also showed a prognostic value. In a series of 122 patients with SMM, Siontis et al. [10] demonstrated that the probability of progression within two years of patients with positive FDG-PET (hyper fixation with or without lytic lesion described on CT) was 75% vs. 30% in patients with negative FDG-PET, without therapy (median progression: 21 months vs. 60 months; *p* < 0.001). Among patients with positive FDG-PET, the probability of progression was 87% at two years when high uptake foci were accompanied by osteolytic lesion (*n* = 16) vs. 61% in the cases without CT lesion (*n* = 9). In a prospective study of 120 patients, Zamagni et al. [11] demonstrated a rate of progression towards MM at two years for patients with positive FDG-PET (FLs without osteolytic involvement in relation to CT) of 58% vs. 33% for patients with negative FDG-PET.

## 4. Therapy Assessment in Symptomatic MM

Obtaining complete metabolic remission (CMR) on FDG-PET exam in an intermediate evaluation before or after ASCT is associated with better survival rates. Bartel et al. [19] showed that the normalization of FDG fixation in FLs after initial chemotherapy courses and prior to ASCT was associated with improved EFS and OS. Confronted with genetic profiles, pre-ASCT CMR bestowed better OS in low risk patients and better EFS in high-risk patients. In 2013, the same team reported the prognostic value of early FDG-PET performed on day 7 of induction therapy, in a series of 302 patients treated according to the same intensive protocol, 277 of whom also had gene expression profiling [25]. The multivariate analysis concluded that more than 3 FLs on FDG-PET on day 7 was associated with lower PFS and OS even in the subgroup of high-risk patients according to genetic profiles. This underlies the value of FDG-PET as a future tool for early corrective therapeutic measures.

In the Italian series of 192 patients, the persistence of a SUVmax >4.2 after induction therapy was associated with a reduced PFS [20]. Three months after ASCT, CMR was obtained in 65% of the patients, with better four-year PFS and OS than those of FDG-PET positive patients. Interestingly, 23% of the patients obtaining complete remission according to conventional criteria were considered as FDG-PET positive. Multivariate analysis showed that post-ASCT FDG-PET status was an independent prognostic factor of PFS. The same team confirmed these findings in 2015, in a series of 282 patients with symptomatic MM undergoing first-line treatment between 2002 and 2012 [26]. Median follow-up was 67 months. After treatment, CMR was obtained in 70% of patients, whereas conventionally defined complete response was observed in 53% of cases. FDG-PET negativization favorably affected PFS and OS. In 12% of patients experiencing relapse, skeletal progression was only detected by systematic FDG-PET during follow-up. Multivariate analysis showed that a SUVmax >4.2 on metabolic imaging after first-line treatment was an independent predictive factor of progression.

The interest of post-ASCT FDG-PET has also been reported in a prospective series of 77 patients evaluated by FDG-PET three months after transplant and every 6–12 months during follow-up [27]. The patients were classified into group 1 (relapse) and group 2 (no relapse). In group 1, the time to relapse was longer when FDG-PET was negative (27.6 months) than when it was positive (18 months) (*p* = 0.05) with a SUVmax inversely correlated with the time to relapse (*p* < 0.01) in PET positive patients. In group 2, 27 patients had a negative FDG-PET and 13 positive but with SUVmax remaining stable in the follow-up.

Finally, the French IMAJEM [18] study confirmed the superiority of FDG-PET as opposed to MRI in the therapeutic evaluation of patients with MM in front-line therapy. This work prospectively compared the value of MRI and FDG-PET realized at diagnosis, after three cycles of induction chemotherapy and before maintenance therapy, in a series of 134 patients treated in the Intergroupe Francophone du Myelome/Dana–Farber Cancer Institute (IFM/DFCI) 2009 clinical trial. FDG-PET normalization after three cycles of induction chemotherapy was associated with a better PFS (*p* = 0.04), as opposed to MRI. Pre-maintenance therapy FDG-PET normalization was equally correlated with improved PFS (*p* < 0.001) and OS (*p* = 0.01), unlike MRI.

Similarly to the evaluation of lymphomas [28], concerted efforts have been made to standardize response assessment for FDG-PET imaging in MM. As described in this review, different groups have reported promising and concordant results. Yet the lack of standard interpretation criteria makes it difficult to draw general guidelines. Several studies mainly relied on semi-quantitative analysis such as SUVmax, while others based their image interpretation on a visual assessment or on both methods. In this context, new interpretation criteria (Italian Myeloma criteria for PETUse; IMPeTUs) were drafted by a group of Italian nuclear medicine experts as a framework that may be useful for harmonizing clinical trials results [29]. Moreover, considering the additive value of imaging-based assessment of minimal residual disease (MRD), the IMWG has recently defined new response categories of MRD negativity [30]. These combine the absence of clonal plasma cells detectable by flow-cytometry or molecular techniques, negative FDG-PET imaging, and a normal heavy/light chain ratio, and probably better represents complete response, to available levels of detection, of malignant cells from all compartments. Further prospective studies are warranted to confirm the validity of these parameters.

## 5. FDG-PET to Detect Relapse

The interest of FDG-PET has also been evaluated in patients with MM suspected of relapse after ASCT [31]. It has been shown in a small series of 37 patients that the absence of FDG avid foci was a prognostic factor associated with better time to relapse and OS (*p* < 0.01). The presence of more than 10 FLs was correlated with lower time to relapse (*p* < 0.01) and OS (*p* < 0.05). The intensity of FDG uptake and the presence of EMD were also correlated with a shorter time to relapse (*p* = 0.037 and *p* = 0.049, respectively). Moreover, the FDG-PET findings led to a change in patients’ management in 30% of cases.

## 6. PET Using Other Radiopharmaceuticals

Certain studies have emphasized the diagnostic interest of new radiotracers in MM. In a pilot study, Cassou-Mounat et al. [13] compared ^18^F-fluorocholine (FCH), a metabolite incorporated into various phospholipids essential in the formation of cell membranes, and FDG for the detection of MM lesions in 21 patients at time of disease relapse or progression. In the 15 patients with countable bone foci, the on-site reader detected 72 FDG foci vs. 127 FCH foci (+76%), and the masked reader 69 FDG foci vs. 121 FCH foci (+75%), both differences being significant. These data suggested that PET performed for suspected relapsing or progressive MM would reveal more lesions when using FCH rather than FDG. Similarly, Lapa et al. [14] prospectively compared the myeloma lesions’ detection sensitivity of FDG and ^11^C-methionine (MET), an amino acid required for protein synthesis, in 43 MM patients for staging or re-staging. MET-PET detected FL in 39 patients (detection rate: 90.7%), whereas 10 patients were missed on FDG-PET (detection rate: 76.7%, *p* < 0.05). MET depicted more FLs in 28 patients (*p* < 0.001). Both FDG and MET uptake correlated significantly with biopsy-proven bone marrow involvement (*p* < 0.001), with MET demonstrating a stronger correlation (SUVmean, *r* = 0.9 vs. *r* = 0.6; SUVmax, *r* = 0.88 vs. *r* = 0.58). Abnormal β-2-microglobulin and free light chain levels correlated with the presence of focal intramedullary lesions detected in MET- or FDG-PET (MET, *p* = 0.006 and *p* = 0.01, respectively; FDG, *p* = 0.02 and *p* = 0.01).

On the other hand, despite a potential theoretical value, discouraging results have been observed regarding the performance of ^18^F-NaF in the assessment of MM. This radiotracer reflects bone remodeling and appears as an interesting imaging method for malignant bone diseases. Yet, as reported in both diagnostic and treatment evaluation [32,33], ^18^F-NaF does not seem to add significant information to FDG-PET in MM patients. 

Theranostic radiopharmaceuticals could also be of interest in MM. CXCR4 is often expressed in high concentration by the monoclonal plasma cells, and a recent study performed in 14 relapsing MM suggested potential of ^68^Ga-Pentixafor, a specific ligand showing a high affinity for CXCR4, offering an excellent contrast in CXCR4-positive patients [16]. CXCR4 can also be targeted by the β-particle-emitters ^177^Lu- or ^90^Y-pentixather for therapeutic purposes, ^68^Ga-Pentixafor allowing for a selection of patients to these therapies in a theranostic approach [34]. Radiolabeled mAbs with radionuclides such as ^64^Cu or ^89^Zr are also considered to select patients before antibody-based therapies [35], and preclinical studies reported feasibility of immuno-PET with ^64^Cu in MM mice models [36].

## 7. Conclusions

FDG-PET constitutes a high performance imaging in symptomatic MM patients to detect medullary and extra-medullary disease at baseline, to assess therapy with prognostic value and to detect relapse after treatment. Yet, FDG-PET interpretation criteria and methods should be standardized for extensive use in clinical practice for symptomatic MM patient management [29]. In SMM, FDG-PET allows for the detection of patients with a high risk of progression towards MM. Pilot studies also reported the interest of innovative radiopharmaceuticals targeting other biomarkers in MM, with potential interest in theranostic approaches.

## References

[1] Röllig C., Knop S., Bornhäuser M. (2015). Multiple myeloma. Lancet.

[2] Rajkumar S.V., Dimopoulos M.A., Palumbo A., Blade J., Merlini G., Mateos M.-V., Kumar S., Hillengass J., Kastritis E., Richardson P. (2014). International Myeloma Working Group updated criteria for the diagnosis of multiple myeloma. Lancet Oncol..

[3] Dimopoulos M.A., Hillengass J., Usmani S., Zamagni E., Lentzsch S., Davies F.E., Raje N., Sezer O., Zweegman S., Shah J. (2015). Role of magnetic resonance imaging in the management of patients with multiple myeloma: A consensus statement. J. Clin. Oncol..

[4] Dimopoulos M., Kyle R., Fermand J.-P., Rajkumar S.V., San Miguel J., Chanan-Khan A., Ludwig H., Joshua D., Mehta J., Gertz M. (2011). International Myeloma Workshop Consensus Panel 3 Consensus recommendations for standard investigative workup: Report of the International Myeloma Workshop Consensus Panel 3. Blood.

[5] Weng W.-W., Dong M.-J., Zhang J., Yang J., Xu Q., Zhu Y.-J., Liu N.-H. (2014). A systematic review of MRI, scintigraphy, FDG-PET and PET/CT for diagnosis of multiple myeloma related bone disease—Which is best?. Asian Pac. J. Cancer Prev..

[6] Lu Y.-Y., Chen J.-H., Lin W.-Y., Liang J.-A., Wang H.-Y., Tsai S.-C., Kao C.-H. (2012). FDG PET or PET/CT for detecting intramedullary and extramedullary lesions in multiple Myeloma: A systematic review and meta-analysis. Clin. Nucl. Med..

[7] Walker R.C., Brown T.L., Jones-Jackson L.B., de Blanche L., Bartel T. (2012). Imaging of multiple myeloma and related plasma cell dyscrasias. J. Nucl. Med..

[8] Salaun P.-Y., Gastinne T., Frampas E., Bodet-Milin C., Moreau P., Bodéré-Kraeber F. (2008). FDG-positron-emission tomography for staging and therapeutic assessment in patients with plasmacytoma. Haematologica.

[9] Fouquet G., Guidez S., Herbaux C., van de Wyngaert Z., Bonnet S., Beauvais D., Demarquette H., Adib S., Hivert B., Wemeau M. (2014). Impact of initial FDG-PET/CT and serum-free light chain on transformation of conventionally defined solitary plasmacytoma to multiple myeloma. Clin. Cancer Res..

[10] Siontis B., Kumar S., Dispenzieri A., Drake M.T., Lacy M.Q., Buadi F., Dingli D., Kapoor P., Gonsalves W., Gertz M.A. (2015). Positron emission tomography-computed tomography in the diagnostic evaluation of smoldering multiple myeloma: Identification of patients needing therapy. Blood Cancer J..

[11] Zamagni E., Nanni C., Gay F., Pezzi A., Patriarca F., Bellò M., Rambaldi I., Tacchetti P., Hillengass J., Gamberi B. (2016). ^18^F-FDG PET/CT focal, but not osteolytic, lesions predict the progression of smoldering myeloma to active disease. Leukemia.

[12] Nanni C., Zamagni E., Cavo M., Rubello D., Tacchetti P., Pettinato C., Farsad M., Castellucci P., Ambrosini V., Montini G.C. (2007). ^11^C-choline vs. ^18^F-FDG PET/CT in assessing bone involvement in patients with multiple myeloma. World J. Surg. Oncol..

[13] Cassou-Mounat T., Balogova S., Nataf V., Calzada M., Huchet V., Kerrou K., Devaux J.-Y., Mohty M., Talbot J.-N., Garderet L. (2016). ^18^F-fluorocholine versus ^18^F-fluorodeoxyglucose for PET/CT imaging in patients with suspected relapsing or progressive multiple myeloma: A pilot study. Eur. J. Nucl. Med. Mol. Imaging.

[14] Lapa C., Knop S., Schreder M., Rudelius M., Knott M., Jörg G., Samnick S., Herrmann K., Buck A.K., Einsele H. (2016). ^11^C-Methionine-PET in Multiple Myeloma: Correlation with Clinical Parameters and Bone Marrow Involvement. Theranostics.

[15] Okasaki M., Kubota K., Minamimoto R., Miyata Y., Morooka M., Ito K., Ishiwata K., Toyohara J., Inoue T., Hirai R. (2015). Comparison of ^11^C-4’-thiothymidine, ^11^C-methionine, and ^18^F-FDG PET/CT for the detection of active lesions of multiple myeloma. Ann. Nucl. Med..

[16] Wester H.J., Keller U., Schottelius M., Beer A., Philipp-Abbrederis K., Hoffmann F., Šimeček J., Gerngross C., Lassmann M., Herrmann K. (2015). Disclosing the CXCR4 expression in lymphoproliferative diseases by targeted molecular imaging. Theranostics.

[17] Zamagni E., Nanni C., Patriarca F., Englaro E., Castellucci P., Geatti O., Tosi P., Tacchetti P., Cangini D., Perrone G. (2007). A prospective comparison of ^18^F-fluorodeoxyglucose positron emission tomography-computed tomography, magnetic resonance imaging and whole-body planar radiographs in the assessment of bone disease in newly diagnosed multiple myeloma. Haematologica.

[18] Moreau P., Attal M., Karlin L., Garderet L., Facon T., Benboubker L., Macro M., Caillot D., Escoffre-Barbe M., Stoppa A.-M. (2014). Prospective Evaluation of MRI and PET-CT at Diagnosis and before Maintenance Therapy in Symptomatic Patients with Multiple Myeloma Included in the IFM/DFCI 2009 Trial. Blood.

[19] Bartel T.B., Haessler J., Brown T.L.Y., Shaughnessy J.D., van Rhee F., Anaissie E., Alpe T., Angtuaco E., Walker R., Epstein J. (2009). F18-fluorodeoxyglucose positron emission tomography in the context of other imaging techniques and prognostic factors in multiple myeloma. Blood.

[20] Zamagni E., Patriarca F., Nanni C., Zannetti B., Englaro E., Pezzi A., Tacchetti P., Buttignol S., Perrone G., Brioli A. (2011). Prognostic relevance of ^18^F FDG PET/CT in newly diagnosed multiple myeloma patients treated with up-front autologous transplantation. Blood.

[21] Haznedar R., Akı S.Z., Akdemir O.U., Ozkurt Z.N., Ceneli O., Yağcı M., Sucak G.T., Unlü M. (2011). Value of ^18^F-fluorodeoxyglucose uptake in positron emission tomography/computed tomography in predicting survival in multiple myeloma. Eur. J. Nucl. Med. Mol. Imaging.

[22] Fonti R., Pace L., Cerchione C., Catalano L., Salvatore B., de Luca S., Pane F., Salvatore M., del Vecchio S. (2015). ^18^F-FDG PET/CT, 99mTc-MIBI, and MRI in the prediction of outcome of patients with multiple myeloma: A comparative study. Clin. Nucl. Med..

[23] Fonti R., Larobina M., del Vecchio S., de Luca S., Fabbricini R., Catalano L., Pane F., Salvatore M., Pace L. (2012). Metabolic tumor volume assessed by ^18^F-FDG PET/CT for the prediction of outcome in patients with multiple myeloma. J. Nucl. Med..

[24] McDonald J.E., Kessler M.M., Gardner M.W., Buros A.F., Ntambi J.A., Waheed S., van Rhee F., Zangari M., Heuck C., Petty N. (2016). Assessment of Total Lesion Glycolysis by ^18^F FDG PET/CT Significantly Improves Prognostic Value of GEP and ISS in Myeloma. Clin. Cancer Res..

[25] Usmani S.Z., Mitchell A., Waheed S., Crowley J., Hoering A., Petty N., Brown T., Bartel T., Anaissie E., van Rhee F. (2013). Prognostic implications of serial 18-fluoro-deoxyglucose emission tomography in multiple myeloma treated with total therapy 3. Blood.

[26] Zamagni E., Nanni C., Mancuso K., Tacchetti P., Pezzi A., Pantani L., Zannetti B., Rambaldi I., Brioli A., Rocchi S. (2015). PET/CT Improves the Definition of Complete Response and Allows to Detect Otherwise Unidentifiable Skeletal Progression in Multiple Myeloma. Clin. Cancer Res..

[27] Nanni C., Zamagni E., Celli M., Caroli P., Ambrosini V., Tacchetti P., Brioli A., Zannetti B., Pezzi A., Pantani L. (2013). The value of ^18^F-FDG PET/CT after autologous stem cell transplantation (ASCT) in patients affected by multiple myeloma (MM): Experience with 77 patients. Clin. Nucl. Med..

[28] Cheson B.D., Fisher R.I., Barrington S.F., Cavalli F., Schwartz L.H., Zucca E., Lister T.A. (2014). Recommendations for Initial Evaluation, Staging, and Response Assessment of Hodgkin and Non-Hodgkin Lymphoma: The Lugano Classification. J. Clin. Oncol..

[29] Nanni C., Zamagni E., Versari A., Chauvie S., Bianchi A., Rensi M., Bellò M., Rambaldi I., Gallamini A., Patriarca F. (2016). Image interpretation criteria for FDG PET/CT in multiple myeloma: A new proposal from an Italian expert panel. IMPeTUs (Italian Myeloma criteria for PET USe). Eur. J. Nucl. Med. Mol. Imaging.

[30] Kumar S., Paiva B., Anderson K.C., Durie B., Landgren O., Moreau P., Munshi N., Lonial S., Bladé J., Mateos M.-V. (2016). International Myeloma Working Group consensus criteria for response and minimal residual disease assessment in multiple myeloma. Lancet Oncol..

[31] Lapa C., Lückerath K., Malzahn U., Samnick S., Einsele H., Buck A.K., Herrmann K., Knop S. (2014). ^18^FDG-PET/CT for prognostic stratification of patients with multiple myeloma relapse after stem cell transplantation. Oncotarget.

[32] Sachpekidis C., Goldschmidt H., Hose D., Pan L., Cheng C., Kopka K., Haberkorn U., Dimitrakopoulou-Strauss A. (2014). PET/CT studies of multiple myeloma using ^18^F-FDG and ^18^F-NaF: Comparison of distribution patterns and tracers’ pharmacokinetics. Eur. J. Nucl. Med. Mol. Imaging.

[33] Sachpekidis C., Hillengass J., Goldschmidt H., Wagner B., Haberkorn U., Kopka K., Dimitrakopoulou-Strauss A. (2017). Treatment response evaluation with ^18^F-FDG PET/CT and ^18^F-NaF PET/CT in multiple myeloma patients undergoing high-dose chemotherapy and autologous stem cell transplantation. Eur. J. Nucl. Med. Mol. Imaging.

[34] Herrmann K., Schottelius M., Lapa C., Osl T., Poschenrieder A., Hänscheid H., Lückerath K., Schreder M., Bluemel C., Knott M. (2016). First-in-Human Experience of CXCR4-Directed Endoradiotherapy with 177Lu- and 90Y-Labeled Pentixather in Advanced-Stage Multiple Myeloma with Extensive Intra- and Extramedullary Disease. J. Nucl. Med..

[35] Kraeber-Bodere F., Bailly C., Chérel M., Chatal J.-F. (2016). ImmunoPET to help stratify patients for targeted therapies and to improve drug development. Eur. J. Nucl. Med. Mol. Imaging.

[36] Halime Z., Frindel M., Camus N., Orain P.-Y., Lacombe M., Chérel M., Gestin J.-F., Faivre-Chauvet A., Tripier R. (2015). New synthesis of phenyl-isothiocyanate *C*-functionalised cyclams. Bioconjugation and ^64^Cu phenotypic PET imaging studies of multiple myeloma with the te2a derivative. Org. Biomol. Chem..

